# A phantom-based analysis for tracking intra-fraction pancreatic tumor motion by ultrasound imaging during radiation therapy

**DOI:** 10.3389/fonc.2022.996537

**Published:** 2022-09-27

**Authors:** Tianlong Ji, Ziwei Feng, Edward Sun, Sook Kien Ng, Lin Su, Yin Zhang, Dong Han, Sarah Han-Oh, Iulian Iordachita, Junghoon Lee, Peter Kazanzides, Muyinatu A. Lediju Bell, John Wong, Kai Ding

**Affiliations:** ^1^ Department of Radiation Oncology, The First Hospital of China Medical University, Shenyang, China; ^2^ Department of Radiation Oncology and Molecular Radiation Sciences, Johns Hopkins School of Medicine, Baltimore, MD, United States; ^3^ Department of Electrical and Computer Engineering, Johns Hopkins University, Baltimore, MD, United States; ^4^ Department of Mechanical Engineering, Johns Hopkins University, Baltimore, MD, United States; ^5^ Department of Computer Science, Johns Hopkins University, Baltimore, MD, United States

**Keywords:** ultrasound image tracking, radiation therapy, respiratory motion, ultrasound image, abdominal

## Abstract

**Purpose:**

In this study, we aim to further evaluate the accuracy of ultrasound tracking for intra-fraction pancreatic tumor motion during radiotherapy by a phantom-based study.

**Methods:**

Twelve patients with pancreatic cancer who were treated with stereotactic body radiation therapy were enrolled in this study. The displacement points of the respiratory cycle were acquired from 4DCT and transferred to a motion platform to mimic realistic breathing movements in our phantom study. An ultrasound abdominal phantom was placed and fixed in the motion platform. The ground truth of phantom movement was recorded by tracking an optical tracker attached to this phantom. One tumor inside the phantom was the tracking target. In the evaluation of the results, the monitoring results from the ultrasound system were compared with the phantom motion results from the infrared camera. Differences between infrared monitoring motion and ultrasound tracking motion were analyzed by calculating the root-mean-square error.

**Results:**

The 82.2% ultrasound tracking motion was within a 0.5 mm difference value between ultrasound tracking displacement and infrared monitoring motion. 0.7% ultrasound tracking failed to track accurately (a difference value > 2.5 mm). These differences between ultrasound tracking motion and infrared monitored motion do not correlate with respiratory displacements, respiratory velocity, or respiratory acceleration by linear regression analysis.

**Conclusions:**

The highly accurate monitoring results of this phantom study prove that the ultrasound tracking system may be a potential method for real-time monitoring targets, allowing more accurate delivery of radiation doses.

## Introduction

The median overall survival of patients with pancreatic cancer is a poor 9 to 15 months (1–4). Only 20% of patients are diagnosed as having resectable disease suitable for surgical treatment (5, 6). The outcomes after chemoradiation for unresectable pancreatic cancer are not ideal as common irradiation doses used in the treatment are not lethal for adenocarcinoma (5). Due to the strong radio resistance of adenocarcinomas, dose escalation is necessary (7). Recently, advanced radiation therapy (RT) techniques of further dose intensification using stereotactic body radiation therapy (SBRT) and intensity modulated radiation therapy (IMRT) have been potential strategies to improve local control (8).

According to previous research, pancreatic tumor motion was greatest in the superior and inferior (SI) direction (9–13). Bussels et al. and Feng et al. observed a larger degree of pancreatic tumor motion to be 24mm 16mm in the SI direction by using dynamic magnetic resonance imaging (MRI) in 12 patients (14, 15). Moreover, Lukas et al. reported that the mean respiration amplitude between inhalation and exhalation was 11 mm with a range of 5-23 mm in the SI direction (5). They also observed the same mean tumor motion with Goldstein et al. in the left and right (LR) and the anterior and posterior (AP) directions (3 mm with a range of 3-7 mm in the LR direction and 4 mm with a range of 3-8 mm in the AP direction) (10). These significant displacements required the use of an additional treatment margin to account for this intra-fraction motion. Previous research demonstrates that the use of a 20-mm margin in the SI direction and a 5-mm margin in the LR and AP directions accounts for respiratory motion without a reference measurement (5). In the published American-French Consensus, it was recommended that the planning target volume (PTV) margin was 15 to 20 mm in the LR and AP directions and 20 to 30 mm in the SI direction by taking into account microscopic spreading, respiratory movements, and set-up margins (16). It is known that the use of increase internal target volume (ITV) was required to account for intra-fraction motion, which is caused by involuntary motion during the treatment process (e.g. Respiration). However, this larger margin will result in delivering greater radiation doses to the area around the tumor, thereby inherently causing toxicities to normal tissues (17–31). In this case, it is not possible to apply dose escalation without margin reduction (32–35).

However, the feasibility of dose escalation with reduced margins necessitates stringent requirements for delivering doses accurately during the treatment process (36). Therefore, the ability to track the pancreatic tumor and identify its position relative to the surrounding normal tissue has become crucial. The adjustment of the treatment fields to target the pancreatic tumor may be implemented and then followed by identifying the location of the pancreatic tumor.

For overcoming this problem, several solutions were proposed and tested in the past. On one hand, a possible solution is to control the beam delivery to follow tumor target movements. D’Souza et al. designed a miniature adaptive robotic couch model built by using two movable platforms (37). This robotic couch is able to move in real-time along with target movements for sparing the unnecessary radiation dose received by surrounding OARs (37). On the other hand, combining varied image modalities with gating techniques, such as MRI (38) and X-ray (39), is another alternative solution for tracking tumor targets during radiation therapy, thereby improving the possibility of implementing dose escalation and reducing the collateral damages.

Ultrasound (US) imaging is a potential method for tracking tumor targets during radiation therapy (40). The obvious advantages of US images are that they are non-ionizing and non-invasive and that they offer non-extra irradiation doses at a low cost (40, 41). Furthermore, US images provide distinct soft-tissue delineation. According to Shinohara and Roach III’s research (42), the US did not rely on the implementation of fiducial markers, which is an invasive process coupled with associated risks. The feasibility of using ultrasound to monitor tumors and guide radiation therapy has previously been studied. Bouchet et al. study (43) demonstrated that ultrasound image reliably transforms image coordinates into treatment coordinates, thereby eliminating the need for probe orientation. Hsu et al (44) found that the motion tracking the performance of a linear array ultrasound probe did not suffer from ultrasound image noise caused by the radiotherapy linear accelerator.

In our previous research, we proposed an arm-bridge system for monitoring intra-fraction real-time movement during pancreas SBRT (45–48). In addition, we introduced and validated an image guidance workflow with a volunteer study. In this study, we aim to further evaluate the accuracy of ultrasound tracking for intra-fraction abdominal targets motion during RT by a phantom-based study.

## Methods and materials

### Input motion data acquisition

Twelve patients with borderline resectable or locally advanced pancreatic cancer (BR/LAPC) who were treated with SBRT at our institution were enrolled in this study approved by the institutional review board. All respiratory cycle data were acquired with a Philips Big Bore 16 slice CT simulator (120 kVp, 1000 mAs/slice, collimation 16 * 1.5 mm, pitch 0.059, rotation time 0.44 s, FOV 600mm, ultrafast recon kernel, 3 mm slice thickness, 3 mm increment, and standard filter) using retrospective helical 4DCT reconstruction software. These displacement points of the respiratory cycle, including amplitude and frequency, were input displacement data transferred to a motion platform (Qusar, Modus QA) to mimic realistic breathing movements in our phantom study.

### Ultrasound system and set-up

Our proposed arm-bridge system was illustrated in our previous paper (45); as a result, we only briefly describe it here.

**Figure 1:**
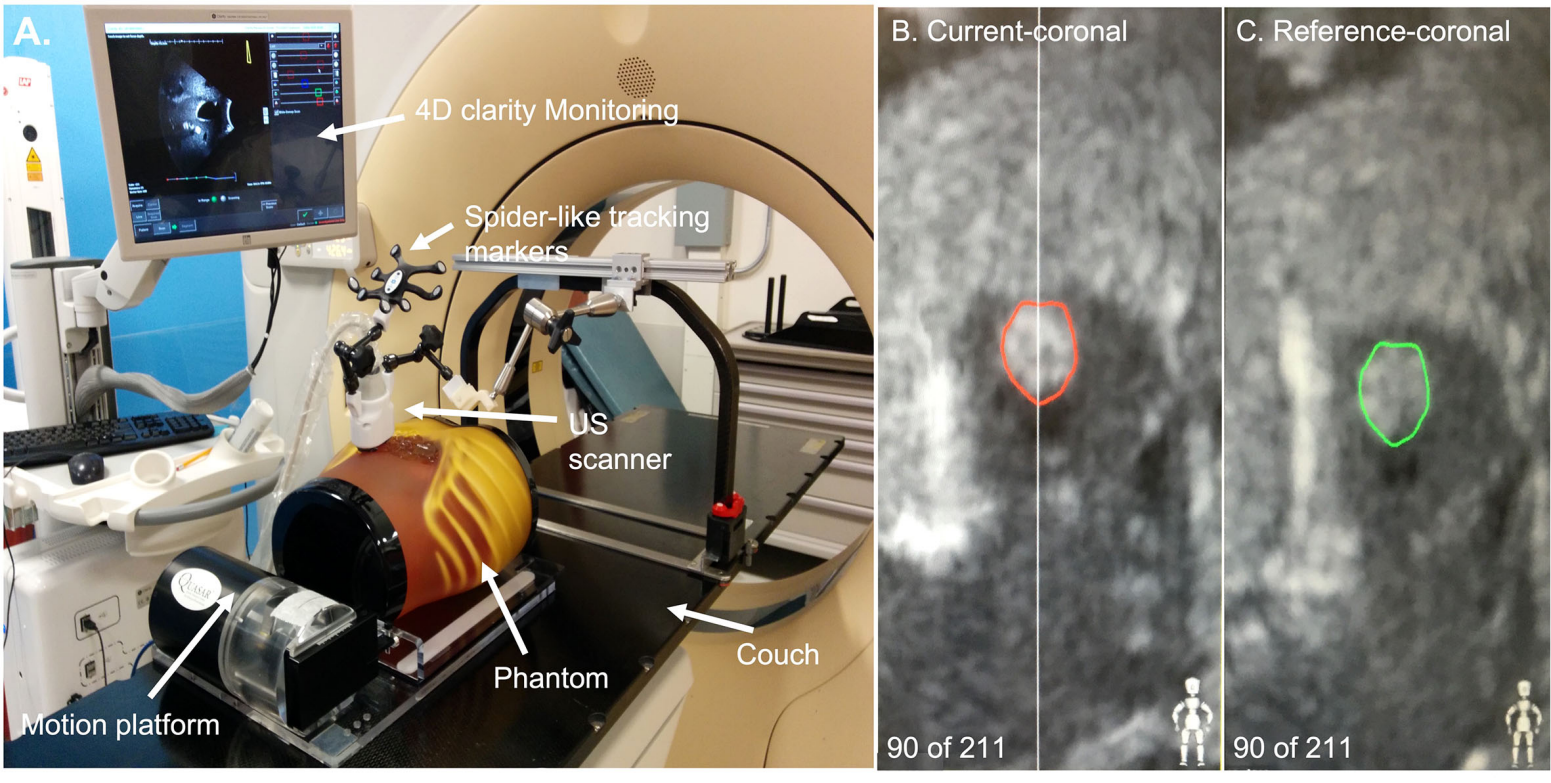
**(A)** The set-up of the ultrasound system. **(B)** An example of tracking the ultrasound image. The red contour was the tracking target in the phantom. **(C)** An example of the original ultrasound image. The green contour was the tracking target contoured manually.

As **Figure 1** showed, an ABDFAN ultrasound phantom (Kyoto Kagaku Co, Japan) implanted with a tantalum fiducials marker was placed in the motion platform. This phantom was fixed to the motion platform. The ground truth of phantom movement was recorded by tracking an optical tracker attached to this phantom. One tumor inside the phantom was considered to be the tracking target. In the evaluation of the results, the monitoring results from the US system were compared with the phantom motion results from the infrared camera (Polaris, NDI). The reason why this phantom surface tracking by infrared camera works in this study is because the ABDFAN ultrasound phantom is a rigid phantom, which is no difference between internal targets movements and phantom surface movements.

The bridge was attached to the top of the couch in a customized bottom rail. The probe we used for this arm-bridge system is a modified mechanically sweeping 4D convex probe (3-7 MHz), which was used for monitoring abdominal targets motion in Clarity Auto-scan (Elekta, Sweden). The probe case was equipped with a spider-like infrared marker and a shorter passive arm. The spider-like marker was monitored by the infrared camera mounted in the room. If the probe position and orientation changed, the ceiling camera was able to detect these changes in real-time by tracking the probe marker. The Clarity ultrasound acquisition system was connected to this probe.

In this study, the motion platform was required to move in SI directions to simulate the breathing movements of the pancreatic tumor during radiation therapy. Therefore, the lateral direction of the ultrasound probe was aligned with this SI direction. In the simulation process, the motion platform started with one position (the original position) and moved to different positions according to the input displacement data of previous patients. In every respiratory cycle, the motion platform went through the original position twice (one in the expiration process and the other one in the inspiration process). The tracking target was contoured manually on a reference-coronal ultrasound image of the phantom obtained when the motion platform was placed at the origin position at the beginning of every simulation (**Figure 1C** reference-coronal). During the tracking process, the real-time ultrasound images were obtained. The tracking targets (red contours in **Figure 1B**) were automatically generated from the intensity-based image-to-image registration method which is able to search for the optimal fit of region of the interest between the real-time images with the reference-coronal images. A detailed explanation of this registration method can be found in Lachaine et al.

### Data analysis

Differences between infrared monitoring motion and US tracking motion were analyzed by calculating the root-mean-square error (RMSE) (Equation 1 shown as follows). Velocity (Equation 2) and acceleration (Equation 3) were calculated for evaluating the reliability and stability of our proposed US tracking method. Linear regression analysis was also used to determine if US tracking motion was in good agreement with infrared monitoring motion. According to previous research, a difference within 2.5 mm is considered a criterion of clinical acceptance (49).


(1) $$RMSE=\sqrt{\frac{\sum_{t=1}^{n}{\left(y_{input}-y_{US}\right)}^{2}}{n}}$$

(mm/s) (2) $$V_{i}=\;\frac{Displacement\;point_{i+1}-\;Displacement\;point_{i}}{Time\;point_{i+1}-\;Time\;point_{i}}$$

Where *Displacement point_i_* represented the displacement (mm) tracked by the infrared camera in *i*th time point, *Time point_i_* is the tracking time point (s) of *i*th sample.


(mm/s). (3) $$a_{i}=\;\frac{V_{i+1}-\;V_{i}}{Time\;point_{i+1}-\;Time\;point_{i}}$$

Where *V_i_* is the velocity (mm/s) of *i*th time point, *Time point_i_* is the tracking time point (s) of *i*th sample.

## Results

**Figure 2:**
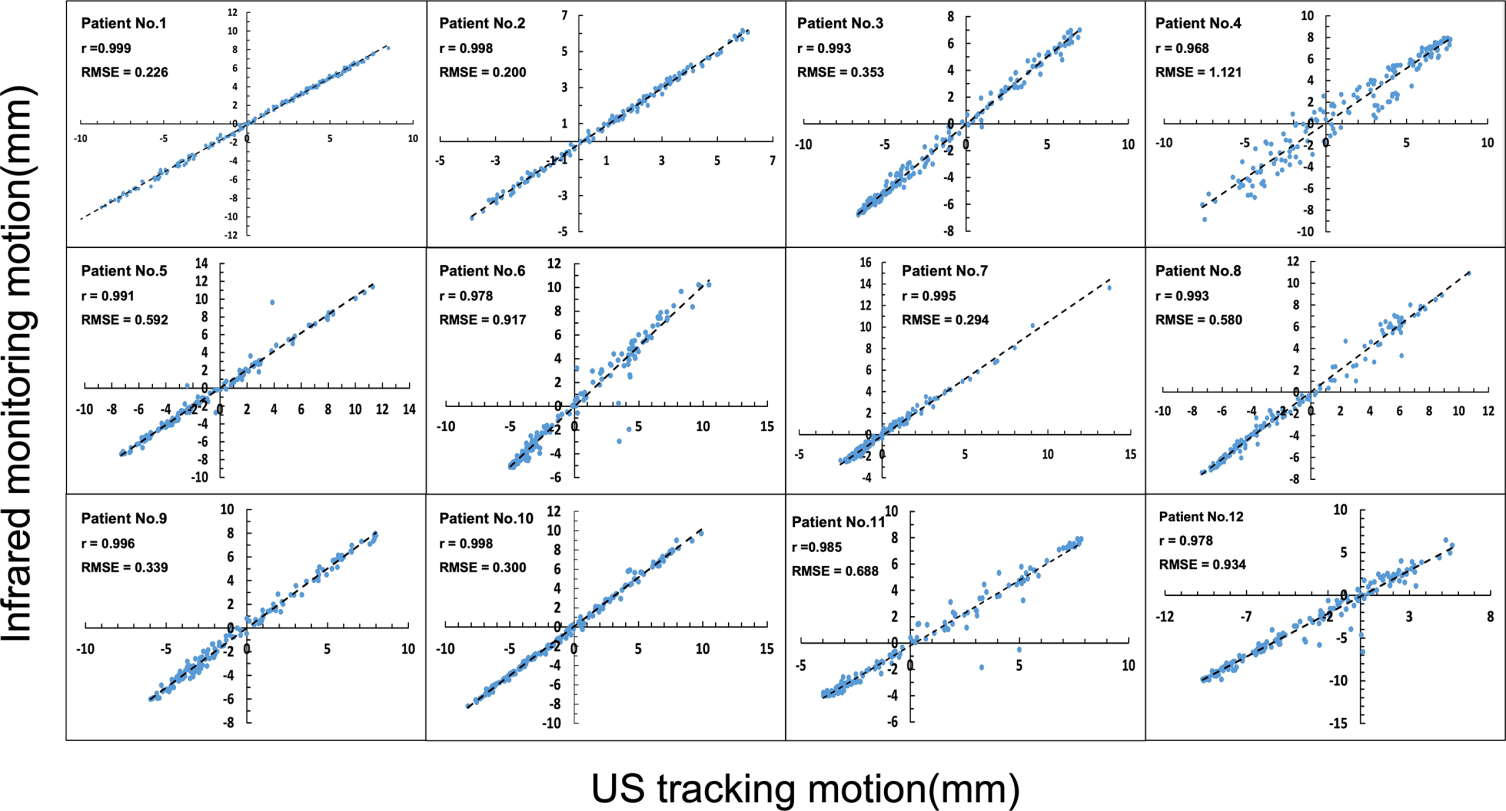
Comparison of infrared monitoring motion and ultrasound tracking motion.

The US tracking motion was plotted against infrared monitoring motion as shown in **Figure 2**. Linear regression analysis resulted in large r values (very close to 1) for all 12 patients. US tracking motion compared well with infrared monitoring motion in most patients. However, patient 4 had a relatively larger RMSE value (RMSE = 1.121). More details about patient 4 will be discussed in the following section.

**Figure 3:**
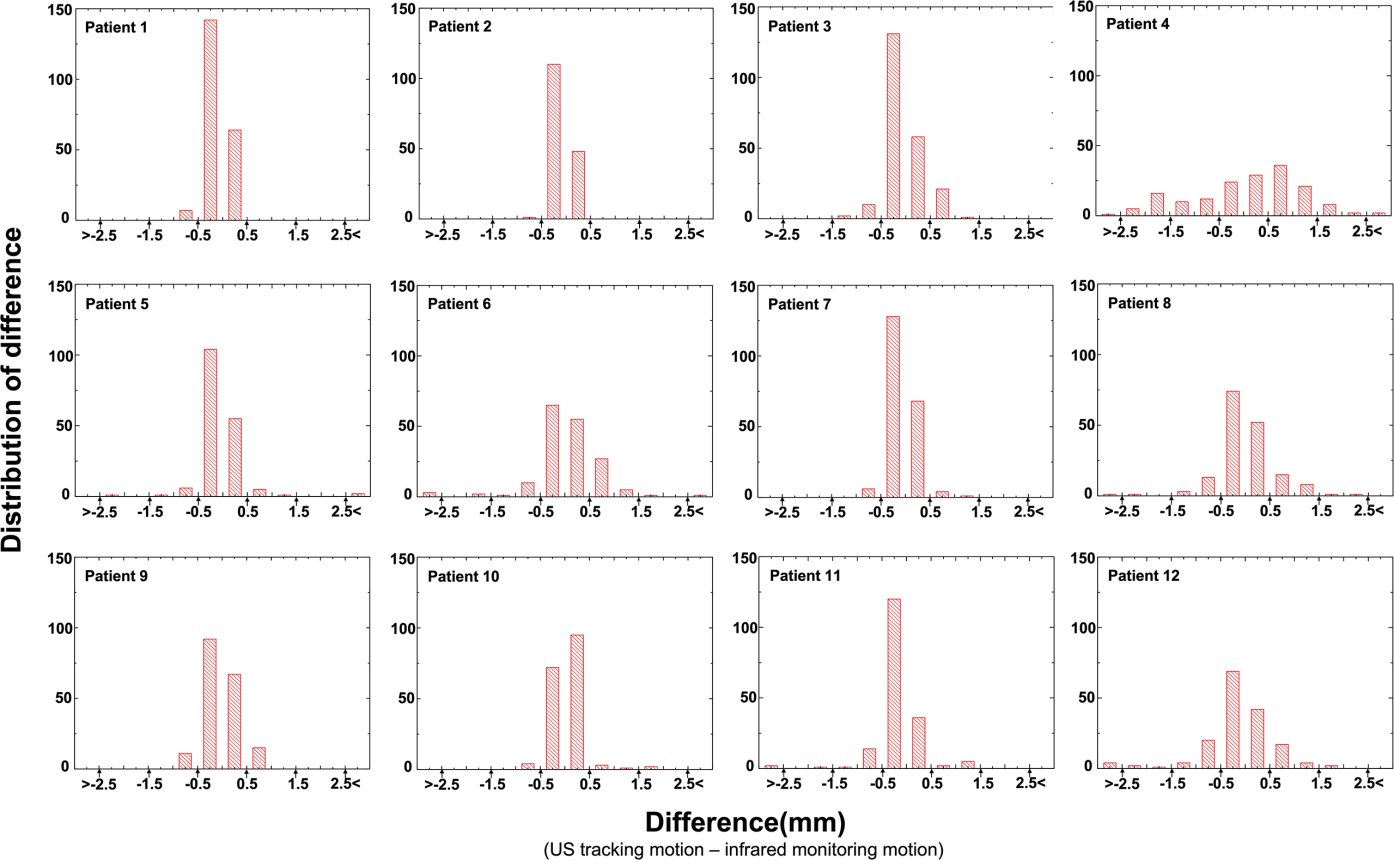
The difference (the difference = US tracking displacement – infrared monitoring motion) histogram showed the number of US tracking motions of every patient.

**Figure 3** provides the distribution of the difference in values for 12 patients. It was found that 82.2% of US tracking motion was within a 0.5 mm difference value and that 0.7% US tracking motion resulted in a failure to track accurately (a difference value > 2.5 mm). This illustrates that the US tracking system is able to monitor abdominal organs with very few tracking errors.

**Figure 4:**
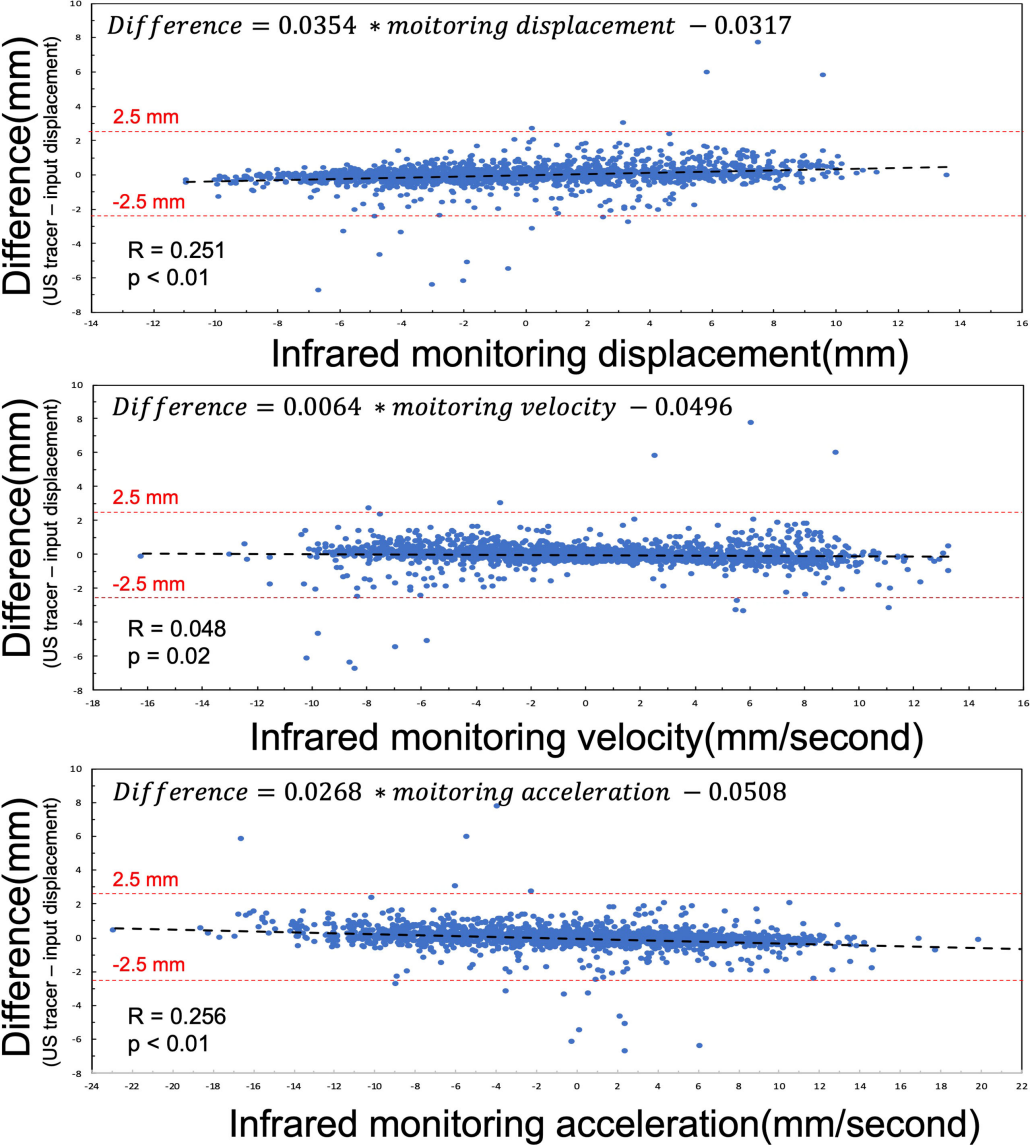
Linear regression analysis between the difference (the difference = US tracking displacement – input displacement) and infrared monitoring motion, infrared monitoring velocity, and infrared monitoring acceleration. The red dash lines represent criterion to estimate tracking results.

The difference between US tracking motion and infrared monitoring motion of 12 patients is summarized in **Figure 4**. Because of the small R value and coefficients, linear regression analysis reveals that these tracking differences do not correlate with respiratory displacements (coefficient is 0.0354, *p<* 0.01, *R* = 0.251), respiratory velocity (coefficient is 0.0064, *p<* 0.02, *R* = 0.048), or respiratory acceleration(coefficient is 0.0268, *p<* 0.01, *R* = 0.256). However, as figure showed, almost difference samples are located within from -2.5 mm to 2.5 mm range randomly. So, in other words, this US tracking system is promising to monitor abdominal organ motion during radiation therapy.

**Figure 5:**
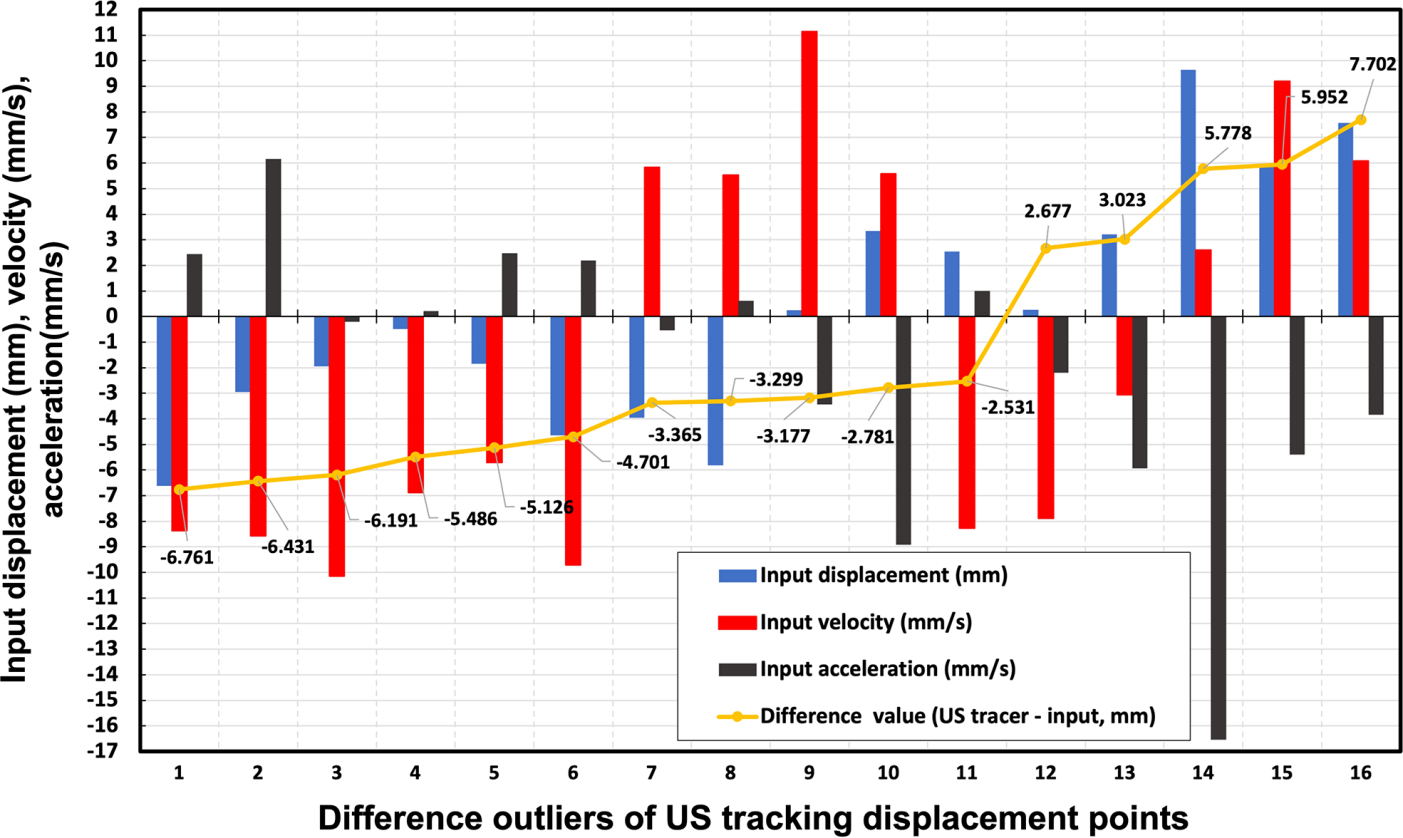
16 difference outliers of US tracking displacement points with the infrared monitoring value, infrared monitoring velocity, and infrared monitoring acceleration. The yellow points represent the difference between US tracking displacement and infrared monitoring values. The blue, red, and black bars are the input displacement, velocity, and acceleration of each tracking point, respectively.

**Figure 5** illustrates 16 difference outliers of US tracking displacement points with the infrared monitoring value, infrared monitoring velocity, and infrared monitoring acceleration. All of these difference outlier absolute values were larger than 2.5 mm, which is considered a criterion for estimating US tracking accuracy. According to the results shown in **Figure 5**, for every outlier, at least one parameter’s (infrared monitoring motion, infrared monitoring velocity, and infrared monitoring acceleration) absolute value exceeded 5. However, in all 2190 input displacement points (the same as US tracking displacement points), the value of infrared monitoring, infrared monitoring velocity, and infrared monitoring acceleration exceeding 5 accounted for 30.6%, 33.8%, and 43.2%, respectively. The difference value exceeding criterion (>2.5 mm or <-2.5 mm) accounted for 0.7%, 1.9%, and 0.5% in these three categories, respectively. On the other hand, infrared monitoring motions also included 70 (accounted for 3.2%) points with an absolute value of three parameters exceeding 5. In these infrared monitoring motions, only 1 (accounting for 0.14%) tracking motion is not within the criterion. A potential explanation will be discussed in a later section.

**Figure 6:**
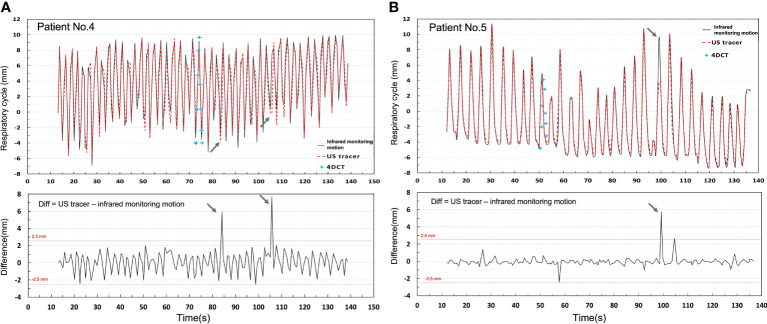
Shows a comparison of infrared monitoring motion, 4DCT, and US tracking displacements of Patients 4 **(A)** and 5 **(B)** with a difference value between infrared monitoring and US tracking displacements.

**Figure 6** depicts a comparison of infrared monitoring, 4DCT, and US tracking displacements of patient 4 (**Figure 6A**) and patient 5 (**Figure 6B**) with a difference value between infrared monitoring and US tracking motion. 4DCT respiratory curve was reconstructed by connecting the centroid point of same target in ten phases. Compared to the 4DCT reconstruct respiratory curve, the US tracking line is able to monitor the target in time. It can be seen that US tracking motion generally agrees with infrared monitoring motion within 2.5 mm in both patients. Moreover, Patient 4 has a larger variation than Patient 5 and the potential reason is that Patient 4 has a relatively shorter respiratory cycle compared to patient 5.

## Discussion

In this phantom study, we investigated the feasibility of ultrasound for monitoring abdominal targets, such as pancreatic cancer, during SBRT. The high monitoring accuracy results prove that the US tracking system may be a potential method for real-time monitoring targets, allowing more accurate delivery of radiation doses. Therefore, the potential benefits of SBRT may be fully realized where the PTV margins that account for target motion may be reduced and the dose-escalation can be achieved.

According to previous research, the motion of the abdominal targets, such as pancreas, caused by respiration is one of the biggest uncertainties in intra-fraction treatment (50). This uncertainty is the main restriction for implementing dose escalation treatment, like SBRT for abdominal tumor targets, such as the pancreatic tumor, and this also is the major motivation of the current phantom study to simulate pancreatic cancer cases. To compensate for this respiratory motion, several intra-fraction monitoring methods were proposed, including X-ray-guided robotic radiation system (Cyberknife from Accuracy) and MRI-guided radiation systems (MRIdian from ViewRay and Unity from Elekta). The Cyberknife system incorporates real-time image guidance and a robot, thereby enabling stereotactic radiosurgery (SRS) and SBRT for different disease sites where motion management is critical. However, due to kV X-ray imaging, every patient absorbs an additional ionizing radiation dose of 0.1 – 0.6 cGy per orthogonal image pair (51). To reduce the imaging dose, the X-ray images are only acquired every 30-60s and thus the treatment beam is likely to miss the target on some occasions (52). Additionally, previous research demonstrated that the management of imaging doses during RT and diagnostic imaging are two different problems (53). The onboard MRI-guided radiation system is a powerful, non-invasive, non-ionizing, and real-time method for tracking soft-tissue targets (54, 55). However, this advanced modality may not be generally available to the community due to its high capital cost. Recently, Han-Oh et al. tested the feasibility of monitoring fiducial markers’ location by using microwave radar (20). Their results proved this microwave radar technology is a potential non-ionizing tumor tracking device during radiation therapy (20). But the feasibility and efficiency of this technology were not yet evaluated in real clinic studies. Compared to these methods, the ultrasound tracking method is an ideal potential solution for monitoring mobile targets at a lower cost, real-time, compatibility when combined with present treatment equipment (56–61). The feasibility of tracking targets in the liver (56) and the prostate (62, 63) has been. Our study found similar agreement to demonstrate that ultrasound tracking is advantageous for tracking SI displacement estimates in abdominal tumors. In addition, such an advantage is expected to improve with new US technology (56).

According to the analysis of all tracking motion points (**Figure 4**), the accuracy of US tracking ability was not restricted by movements, respiration rate, or respiration acceleration. In terms of imaging frame rate, monitoring pancreatic targets by the US has a reasonable temporal resolution. Regarding whether the tracking accuracy is always within clinical requirements (26), however, we found that Patient 4 has the largest RMSE (RMSE = 1.121) and the lowest r value (r = 0.968) as seen in **Figure 2**. This patient has a larger variation of the difference between input and tracking displacements, even though all these errors fall within 2.5mm. The likely reason these relatively unstable tracking results occurred is that this patient has the shortest respiratory cycle (1.37s). On the contrary, the other 11 patients’ respiratory cycles lie between 2.93s and 8s. Due to the limited number of patients’ respiratory cycles, we did not find statistical significance in the relationship between the respiratory cycle and RMSE. More patients’ respiratory data are needed to include in a future study for further testing the tracking ability.

The other limitation of the current study is that we did not address intra-fraction pancreatic target movement in the LR or the AP directions. While LR and AP motions are generally smaller in magnitude and less frequent than SI motions, these displacements may need to be taken into consideration when exploring the use of small PTV margins or dose escalation. In addition, the terrible contrast of pancreatic tumor, or other soft tissue in the traditional ultrasound image is because unique geometric anatomy, such as pancreas located behind stomach and duodenum. But our mechanical robotic arm could fix the ultrasound transducer, thereby pressing the patients’ body for getting optimal contrast. Plus, our proposed workflow and methods could be used to track other abdominal targets, such as liver tumor.

Furthermore, in this study the internal targets’ movements were simulated by shifting the whole rigid phantom on the motion platform. A deformable motion phantom that can simulate both target motion and surface deformation would be ideal. In addition, in this study, the ultrasound transducer has to be placed and fixed by using a robotic arm or a passive surgical arm (41, 45). To maintain the constant contact with the body surface for ultrasound motion tracking, additional force from the probe and fixation holder weight and the locking mechanism is needed and they can cause body surface and internal anatomical deformation. An ultrasound transducer such as the flexible ultrasound probe in recent development with distributed force or pressure along the body surface will be desired.

## Conclusion

The high monitoring accuracy results of this phantom study prove that the US tracking system may be a potential method for real-time monitoring targets, allowing more accurate and conformal delivery of radiation doses. These findings demonstrate that the US tracking system, which is portable, non-ionizing, and non-invasive, can be used as an alternative method to CT, MRI, and marker-based imaging methods for intra-fraction motion tracking.

## Data availability statement

The raw data supporting the conclusions of this article will be made available by the authors, without undue reservation.

## Ethics statement

The studies involving human participants were reviewed and approved by the Johns Hopkins Medicine Institutional Review Boards. The authors are accountable for all aspects of the work in ensuring that questions related to the accuracy or integrity of any part of the work are appropriately investigated and resolved.

## Author contributions

The study was designed by TJ, ZF, and KD. All authors participated in collecting data. TJ, ZF, and KD prepared the manuscript and contributed to data analysis and interpretation. All authors contributed to the article and approved the submitted version.

## Funding

This work was supported,in part, by grants from the National Institutes of Health (NCI RO1 CA161613), Elekta to JW, and Beijing Eastraycloud Technology and Sciences Co. Ltd to TJ.

## Conflict of interest

The authors declare that the research was conducted in the absence of any commercial or financial relationships that could be construed as a potential conflict of interest.

## Publisher’s note

All claims expressed in this article are solely those of the authors and do not necessarily represent those of their affiliated organizations, or those of the publisher, the editors and the reviewers. Any product that may be evaluated in this article, or claim that may be made by its manufacturer, is not guaranteed or endorsed by the publisher.

## Abbreviations

RT, radiation therapy; SBRT, stereotactic body radiation therapy; IMRT, intensity modulated radiation therapy; SI, superior and inferior; LR, left and right; AP, anterior and posterior; US, ultrasound; MRI, magnetic resonance imaging; PTV, planning target volume; BR/LAPC, borderline resectable or locally advanced pancreatic cancer; CT, computed tomography; 4DCT, four-dimensional computed tomography; FOV, field of view; RMSE, root-mean-square error; SRS, stereotactic radiosurgery.
